# The complexity of nicotinamide adenine dinucleotide (NAD), hypoxic, and aryl hydrocarbon receptor cell signaling in chronic kidney disease

**DOI:** 10.1186/s12967-023-04584-8

**Published:** 2023-10-09

**Authors:** Colleen S. Curran, Jeffrey B. Kopp

**Affiliations:** 1 National Heart Lung and Blood Institute, NIH, BG 10 RM 2C135, 10 Center Drive, Bethesda, MD 20814 USA; 2 Kidney Disease Section, NIDDK, NIH, Bethesda, MD USA

**Keywords:** Inflammation, Hypoxia-inducible factor (HIF), Glycolysis, Nicotinamide adenine dinucleotide (NAD), Aryl hydrocarbon receptor (AHR), Sirtuin, Poly-ADP ribose polymerase** (**PARP), NF-κB, Fibrosis

## Abstract

Early-stage detection of chronic kidney diseases (CKD) is important to treatment that may slow and occasionally halt CKD progression. CKD of diverse etiologies share similar histologic patterns of glomerulosclerosis, tubular atrophy, and interstitial fibrosis. Macro-vascular disease and micro-vascular disease promote tissue ischemia, contributing to injury. Tissue ischemia promotes hypoxia, and this in turn activates the hypoxia-inducible transcription factors (HIFs). HIF-1α and HIF-2α, share a dimer partner, HIF-1β, with the aryl hydrocarbon receptor (AHR) and are each activated in CKD and associated with kidney cellular nicotinamide adenine dinucleotide (NAD) depletion. The Preiss-Handler, salvage, and de novo pathways regulate NAD biosynthesis and gap-junctions regulate NAD cellular retention. In the Preiss-Handler pathway, niacin forms NAD. Niacin also exhibits crosstalk with HIF and AHR cell signals in the regulation of insulin sensitivity, which is a complication in CKD. Dysregulated enzyme activity in the NAD de novo pathway increases the levels of circulating tryptophan metabolites that activate AHR, resulting in poly-ADP ribose polymerase activation, thrombosis, endothelial dysfunction, and immunosuppression. Therapeutically, metabolites from the NAD salvage pathway increase NAD production and subsequent sirtuin deacetylase activity, resulting in reduced activation of retinoic acid-inducible gene I, p53, NF-κB and SMAD2 but increased activation of FOXO1, PGC-1α, and DNA methyltransferase-1. These post-translational responses may also be initiated through non-coding RNAs (ncRNAs), which are additionally altered in CKD. Nanoparticles traverse biological systems and can penetrate almost all tissues as disease biomarkers and drug delivery carriers. Targeted delivery of non-coding RNAs or NAD metabolites with nanoparticles may enable the development of more effective diagnostics and therapies to treat CKD.

## Introduction

**Fig. 1:**
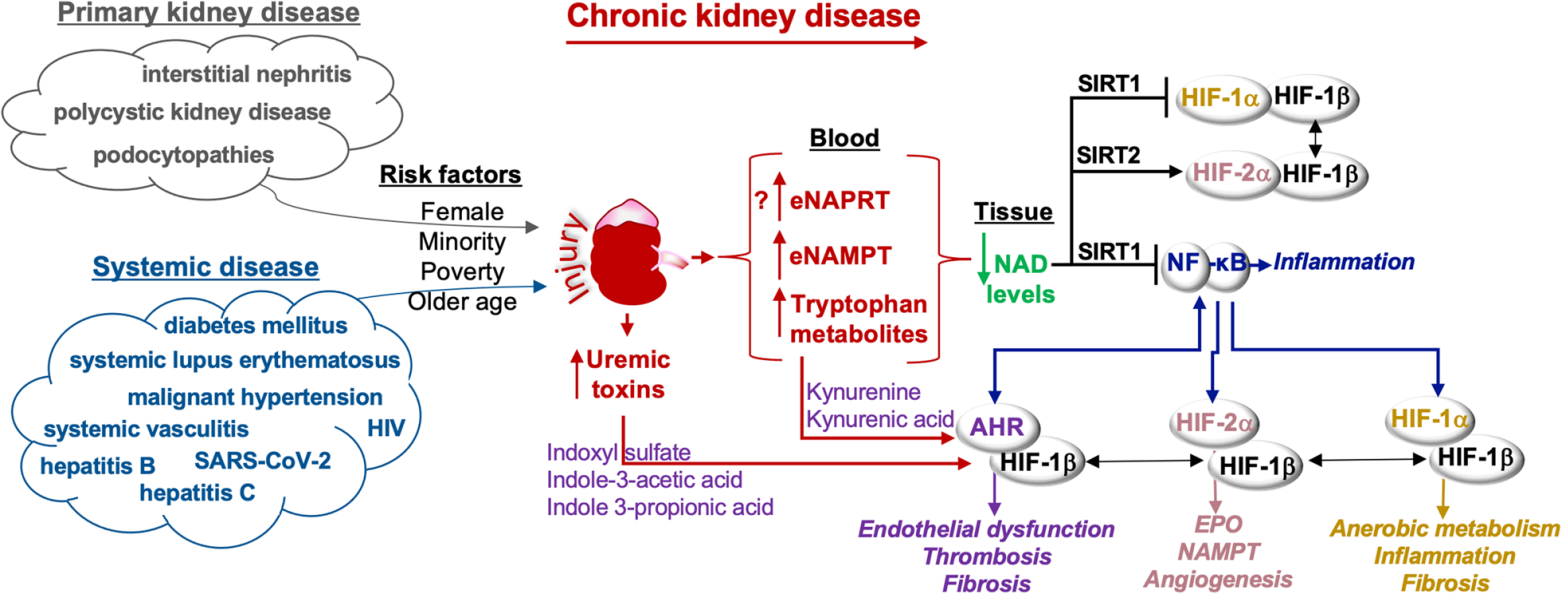
**CKD promotes hypoxic and xenobiotic cell signals and depletes tissue levels of NAD**. CKD is caused by primary or systemic diseases and is prevalent among older individuals, women, ethnic minorities and those living in poverty. Independent of the initial cause of injury, CKD progression is irreversible. Injury induces damage, culminating in the production of uremic toxins and the release of enzymes and molecules required for NAD production into the blood. NAD is a substrate for sirtuins (SIRTs) that can inhibit the activity of hypoxia-inducible transcription factor (HIF)-1α, aryl hydrocarbon receptor (AHR), and nuclear factor (NF)-κB but promote HIF-2α stability. AHR exibits crosstalk with NF-κB. HIFs share a dimer partner with AHR to activate genes. In the de novo pathway of NAD biosynthesis, tryptophan metabolites are released into the blood and like uremic toxins, activate AHR. Nicotinamide phosphoribosyltransferase (NAMPT) and nicotinic acid phosphoribosyltransferase (NAPRT) are rate-limiting enzymes in the NAD salvage pathway and Preiss-Handler pathway, respectively. Extracellular NAMPT (eNAMPT) and NAPRT (eNAPRT) can be released into the blood and activate toll-like receptor (TLR)-4. In CKD, plasma levels of eNAMPT are elevated. Whether eNAPRT is similarly released to deter NAD production in tissue is not known. NF-κB promotes inflammation and the transcription of HIFs and AHR

Chronic kidney disease (CKD) affects more than 10% of the global population and is a leading cause of mortality. CKD is caused by primary and systemic diseases and is prevalent among older individuals, women, racial/ethnic minorities and those living in poverty [1] (Fig. 1). Hypertension, diabetes, obesity, and dyslipidemia are primary risk factors that increase rates of cardiovascular disease and mortality in patients with CKD [2]. Drugs such as angiotensin-converting enzyme inhibitors, angiotensin II receptor blockers, metformin, and low- to moderate-dose statins may reduce the risk of cardiovascular disease. Kidney injury may alter glomerular filtration, tubular reabsorption and secretion processes and subsequently, drug efficacy and toxicity [3].

Glomerular hyperfiltration or increased natriuresis can be signs of renal dysfunction that reflect damage to the glomerular filtration barrier. This ultrafiltration process consists of a fenestrated endothelium, glomerular basement membrane, and podocytes that function concurrently through cell junction intercellular communication [4]. Gap junctions are membrane channels that allow for the direct cytoplasmic exchange of ions and low-molecular weight metabolites (< 1 kDa) between adjacent cells [5]. The gap junction protein connexin 43 is dysregulated in CKD and blocking this molecule is a suggested therapy in treating the disease [4, 5]. Connexin 43 is also a hemichannel that mediates equilibrative transport of nicotinamide dinucleotide (NAD) from the cytosol to the extracellular microenvironment [6], suggesting that connexin 43 is also associated with cellular NAD homeostasis.

CKD typically progresses through five stages, with kidney failure being the most severe. Because the early stages of CKD may lack changes in the glomerular filtration rate and therefore remain undetected until advanced stages, additional diagnostic techniques are being investigated to include nanoparticles [7], which may also be employed in CKD therapies for targeted delivery of molecules based on size, charge, or hydrophobicity [8]. Advanced CKD requires kidney replacement therapy and when possible, kidney transplant [9]. Complications of dialysis and allograft failure increase morbidity, mortality, and healthcare costs [10, 11], indicating that better strategies are needed to prevent, slow, or halt progressive loss of kidney function in patients with CKD.

CKD progression is irreversible (although some therapies may slow or even halt CKD) and is associated with progressive fibrosis, loss of peritubular capillaries, reduced peritubular capillary blood flow, renal anemia, and ultimately renal hypoxia [12]. Under hypoxic conditions, the hypoxia-inducible transcription factor (HIF)-prolyl hydroxylase domain (PHD) pathway is active. Three PHD isoforms (PHD-1, 2, 3) add hydroxyl groups to HIFs. Of the three HIF isoforms (HIF-1α, 2α, 3α), the status of only HIF-1α and HIF-2α has been extensively characterized in CKD [13].

**Fig. 2:**
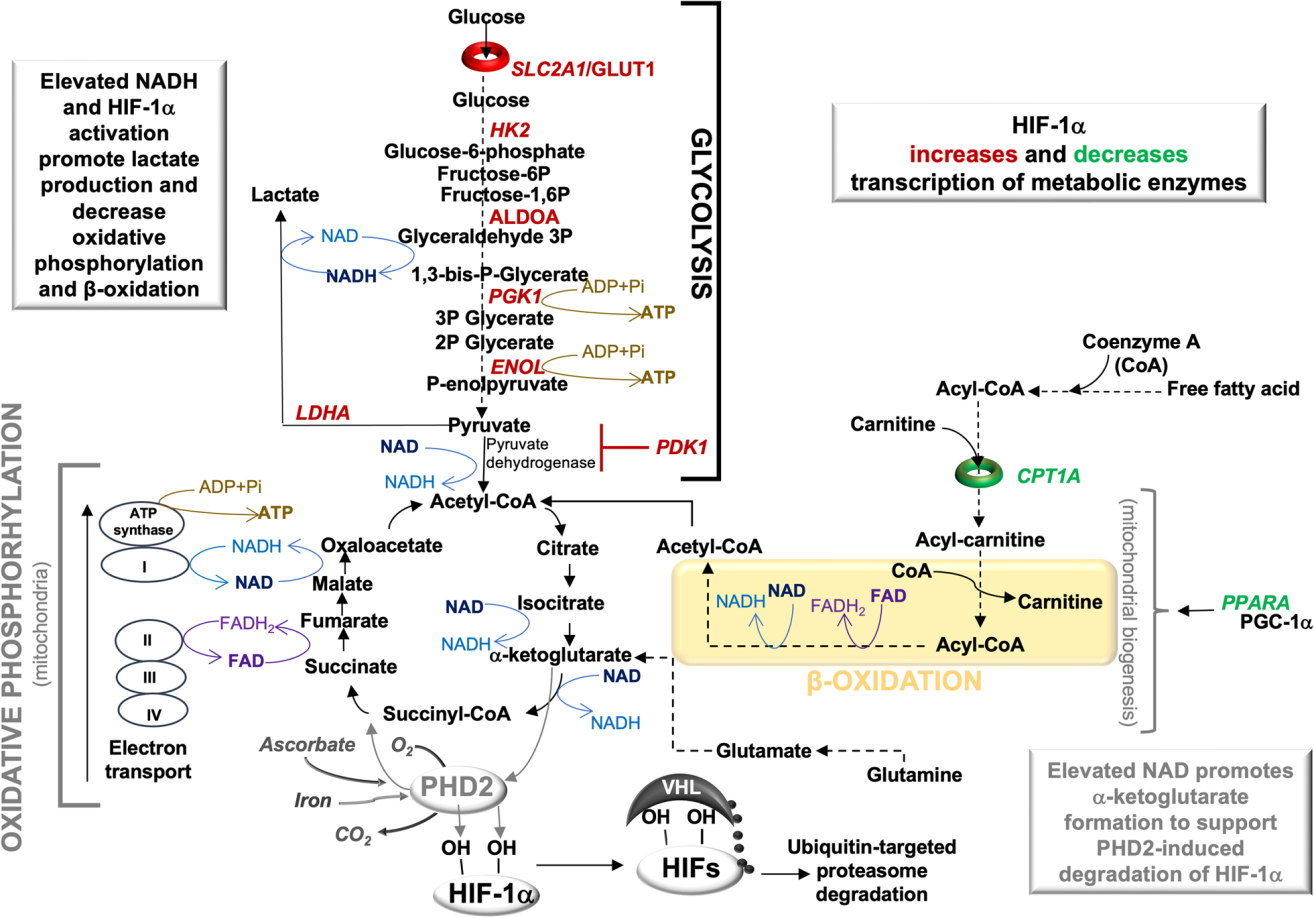
**NAD and hypoxic metabolism**. Inflammation, reactive oxygen species, and/or hypoxia promote the stabilization of hypoxia-inducible transcription factor (HIF)-1α. A dimer, composed of HIF-1α and HIF-1β, activates glycolytic genes, including *SLC2A1/GLUT1* (glucose transporter), *HK2* (hexokinase 2), *ALDOA* (aldolase), *PGK1* (phosphoglycerate kinase-1), *ENOL* (enolase), *LDHA* (lactate dehydrogenase A), and *PDK1* (pyruvate dehydrogenase kinase 1). These gene products redirect pyruvate catabolism in the mitochondria to the production of lactate. Further, the HIF-1α:HIF-1β heterodimer inhibits the transcription of fatty acid genes associated with β-oxidation. *CPT1A* (carnitine palmitoyltransferase 1A) catalyzes the transfer of acyl groups into the mitochondria. *PPARA* (peroxisome proliferator activated receptor alpha) is a cofactor to PGC-1 α (Peroxisome proliferator-activated receptor-gamma coactivator-1 alpha) in mitochondrial biogenesis. Redox reactions involving oxidized NAD (nicotinamide adenine dinucleotide) or FAD (flavin adenine dinucleotide) and their reduced forms (NADH, FADH) promote each form of metabolism, resulting in the production of ATP (adenosine triphosphate) from ADP (adenosine diphosphate) and Pi (inorganic phosphate). Acetyl-CoA, formed from glycolysis or β -oxidation, fuels the citric acid cycle and the electron transport chain. NAD reduction reactions are important for α-ketoglutarate production, which is also formed through glutaminolysis and the production of glutamate. Iron, oxygen, ascorbate, and α-ketoglutarate support the activation of prolyl-hydroxylase (PHD)-2, which adds hydroxyl groups to HIF-1α and promotes HIF-1α binding to the von Hippel-Lindau (VHL) E3 ubiquitin ligase. VHL adds ubiquitin to HIF-1α, targeting HIF-1α for degradation by the proteasome.

In tissue normoxia, PHD-1 and PHD-2 exhibit respective substrate preference for HIF-2α and HIF-1α [14], resulting in HIF hydroxylation, ubiquitination and degradation [13, 15]. HIF-1α is primarily associated with metabolic programming (Fig. 2) whereas HIF-2α regulates tissue revascularization [16], the production of erythropoietin [14], and the NAD enzyme that also functions as a cytokine, nicotinamide phosphoribosyltransferase (NAMPT) [17]. Because renal anemia is a common complication in patients with CKD, pharmaceutically activating the HIF-PHD pathway to promote erythropoietin production is being considered as a therapy [18]. However, the HIF-PHD pathway exhibits crosstalk with the aryl hydrocarbon receptor (AHR) through a shared dimer partner, HIF-1β. Each of these pathways contribute to kidney injury and renal tubulointerstitial fibrosis in progressive kidney disease [13, 19, 20], indicating that HIF-PHD downstream responses are complex (Figs. 1 and 2).

**Fig. 3:**
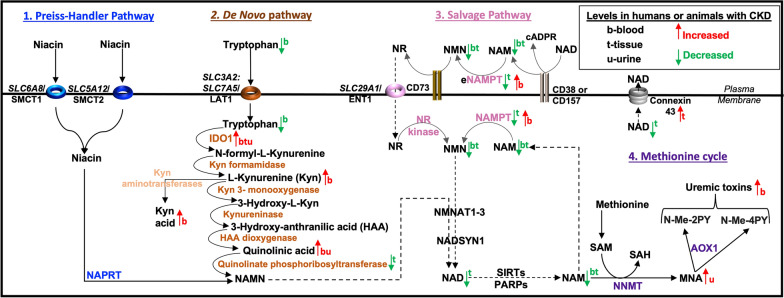
**NAD metabolism in CKD**. (1) Monocarboxylate transporters facilitate niacin transport into cells and in the kidney, transport occurs primarily through sodium-coupled monocarboxylate transporters (SMCT1, SMCT2). Nicotinic acid phosphoribosyltransferase (NAPRT) is the rate-limiting enzyme that forms nicotinic acid mononucleotide (NAMN). Conversion of NAMN to NAD (nicotinamide adenine dinucleotide) occurs through nicotinamide mononucleotide adenylyltransferases (NMNAT1-3 (2) L-type amino acid transporter (LAT1) is a heterodimer whose protein constituents are encoded by *SLC3A2* and *SLC7A5* genes. LAT1 transports tryptophan into cells. Indoleamine 2, 3-dioxygenase 1 (IDO1) is the rate-limiting enzyme. A series of additional enzymatic reactions produces NAMN, which is also formed in the Preiss-Handler pathway (as shown). Kynurenine aminotransferases generate kynurenic acid instead of NAD pathway metabolites. Conversion of NAMN to NAD occurs through NMNAT1-3 and NAD synthetase 1 (NADSYN1). (3) NAD is catabolized by NADases (CD38, CD157), resulting in the release of nicotinamide (NAM) and cyclic ADP ribose (cADPR) from the cell. Extracellular nicotinamide phosphoribosyltransferase (eNAMPT) transforms NAM into nicotinamide mononucleotide (NMN). Extracellular NMN can be transformed to nicotinamide riboside (NR) by CD73, and NR is transported into the cell through an equilibrative nucleoside transporter 1 (ENT1) encoded by *SLC29A1*. Inside the cell, an NR kinase transforms NR into NMN. NAMPT also transforms NAM into NMN. Conversion of NMN to NAD occurs through the actions of NMNAT1-3 and NADSYN1. (4) NAD consuming enzymes (SIRTs, PARPs) produce NAM. Nicotinamide N-methyltransferase (NNMT) catalyzes the transfer of a methyl group from S-adenosylmethionine (SAM) to NAM, producing S-adenosylhomocysteine (SAH) and N1-methylnicotinamide (MNA). Aldehyde oxidase-1 (AOX1) forms n-methyl-2-pyridone-5-carboxamide (N-Me-2PY) and n-methyl-4-pyridone-3-carboxamide (N-Me-4PY), which are characterized as uremic toxins. Enzymes and molecules altered in CKD are highlighted (see text for additional details)

Complications of progressive disease can include renal anemia [18], insulin resistance [21], kidney fibrosis [13], inflammation [22], tubular necrosis [23], and endothelial dysfunction [24]. The diversity of responses in CKD can be associated with a muti-faceted metabolic factor, nicotinamide adenine dinucleotide (NAD), which is an enzyme co-factor in redox reactions or a substrate consumed by sirtuins (SIRTs) and poly-ADP ribose polymerases (PARPs). During hypoxia, elevated levels of the reduced form of NAD (NADH) are produced compared to the oxidized form (NAD) [25] (Fig. 2). NAD is generated from dietary niacin (via the Preiss-Handler pathway) and from dietary tryptophan (via the de novo pathway). The addition of an amide to niacin forms nicotinamide (NAM), which is also a by-product of NAD consumption. The recycling of NAD precursors (e.g., NAM, nicotinamide mononucleotide, and nicotinamide riboside) occurs through the NAD salvage pathway [26]) (Fig. 3).

**Fig. 4:**
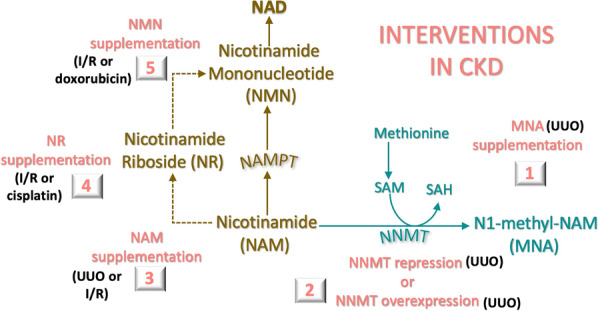
**CKD interventions associated with altered NAD metabolism in mouse models**. (1) Gavage treatment with N1-methylnicotinamide (MNA) in a unilateral ureteral obstruction (UUO) murine model ameliorated renal fibrosis. (2) Renal fibrosis was ameliorated in UUO models by nicotinamide n-methyltransferase (NNMT) deficiency or injections of NNMT-expressing plasmids. (3) Intraperitoneal administration of nicotinamide reduced fibrosis and injury in UUO and ischemia and reperfusion (I/R) models, respectively. (4) Oral or intraperitoneal nicotinamide riboside ameliorates murine I/R or cisplatin induced injury and fibrosis. (5) Intraperitoneal administration of nicotinamide mononucleotide reduced fibrosis and inflammation and improved kidney function in murine I/R and Adriamycin (doxorubicin) injury models

In various animal models of CKD, kidney tissue NAD levels are reduced [27, 28] and treatment with NAD metabolites stabilizes or improves kidney function and pathology [29–31] (Fig. 4). Consequently, clinical trial investigations of NAD metabolites in CKD have been carried out (niacin: NCT00852969, nicotinamide riboside: NCT04818216) and are ongoing (nicotinamide riboside: NCT04040959, Nicotinamide: NCT04589546). The functions of NAD metabolites in CKD are therefore important to understanding disease progression and the therapeutic response.

In this review, we examine NAD biosynthesis pathways and their integrated networks involving hypoxic and xenobiotic cell signals, as both contributors to and consequences of CKD. We offer future perspectives for increased understanding and exploitation of these networks and therapies involving NAD metabolites.

### Niacin

Niacin (nicotinic acid, vitamin B3) is a water-soluble vitamin identified in 1937 as treatment for pellagra, a niacin deficiency disease associated with dermatitis, dementia, diarrhea, and in severe cases, death [32]. Today, niacin is also a therapeutic option for atherosclerosis and dyslipidemia due to its effects in lowering levels of low-density lipoprotein (LDL) cholesterol, reducing triglycerides and lipoprotein (a) levels, and increasing high-density lipoprotein (HDL) cholesterol levels [33]. These beneficial niacin responses are complicated by side effects, including hepatotoxicity [34] and increased prostaglandin production that leads to prostaglandin-induced vasodilation of small capillaries and cutaneous flushing [35]. Pharmaceutically adjusting the release rate of niacin that is absorbed in the intestines and delivered to the liver (e.g., immediate-release, extended-release, timed-release, controlled-release) may mitigate adverse events associated with niacin therapy [36]. However, the varied responses in patients [37, 38] and in dissolution profiles of commercially available niacin formulations [36], indicate that further study of the molecules function in human health is warranted.

Niacin is metabolized via two pathways, the conjugative pathway and non-conjugative (Preiss-Handler) pathway. The conjugative pathway is active only when the Preiss-Handler pathway is saturated and involves the conjugation of free niacin to glycine, forming the metabolite, nicotinuric acid. Niacin, nicotinuric acid, and additional niacin conjugates activate hydroxy-carboxylic acid receptors (HCARs) [39]. Niacin receptors are most strongly expressed on adipocytes [40].

### The niacin conjugation pathway and insulin

**Fig. 5:**
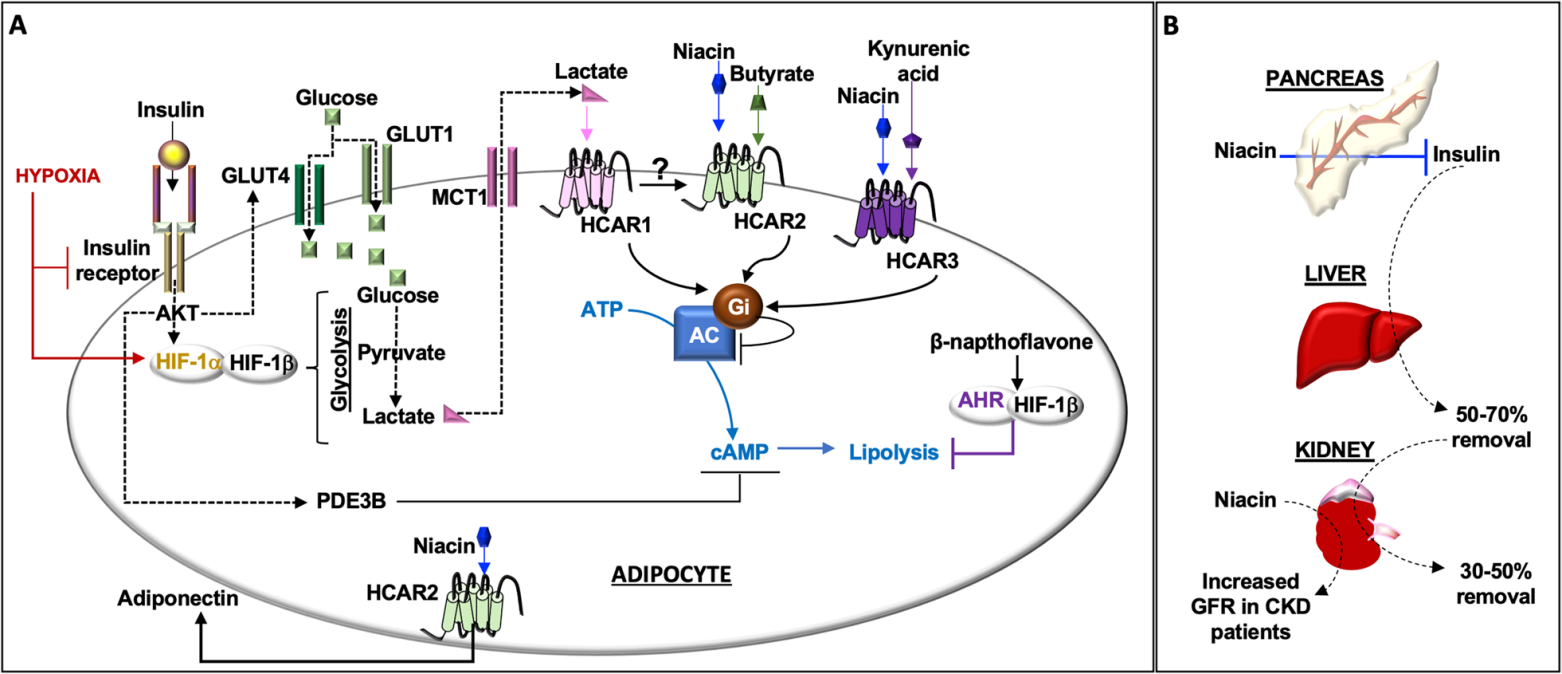
**Niacin and insulin signaling**. (**A**) Insulin binds its receptor on adipocytes and this receptor activates the AKT serine/threonine kinase (also known as protein kinase B). AKT generates downstream signals, including PDE3B (phosphodiesterase 3B) and HIF-1α (hypoxia inducible transcription factor-1α). HIF-1α cell signals promote glycolysis, lactate production, and the release of lactate from cells through the transporter, MCT1 (SLC16A1/monocarboxylate transporter 1). Hypoxia activates HIF-1α and its dimer partner HIF-1β but inhibits insulin receptor activation. HCARs (hydroxy-carboxylic acid receptors) are G-protein-coupled receptors. HCARs bind distinct ligands and induce Gi (inhibitory) signals to AC (adenylate cyclase) that catalyzes the formation of cAMP (cyclic adenosine monophosphate) from ATP (adenosine triphosphate). The function of HCAR1 may depend, in part, on the presence of HCAR2. PDE3B, Gi, and β-napthoflavone activated aryl hydrocarbon (AHR) signals antagonize lipolysis. Niacin also induces serum adiponectin production through HCAR2. (**B**) Insulin is produced by pancreatic islet beta-cells and circulates through the liver and kidney, as well as other tissues. Niacin inhibits insulin production and increases glomerular filtration rates (GFR) in CKD

All HCARs inhibit lipolysis through distinct ligands. HCAR1/GPR81 binds the HIF-1α downstream product, lactate [41]. HCAR2/GPR109A binds niacin or butyrate [41, 42]. HCAR3/GPR109B binds niacin or the AHR ligand, kynurenic acid [41, 43]. Activation of AHR by β-napthoflavone also inhibits lipolysis [44], highlighting crosstalk among these pathways, including a role for insulin, the most potent inhibitor of lipolysis [45] (Fig. 5).

Niacin activation of HCAR2 in rat INS-1 insulinoma cells inhibits insulin secretion [46]. In a murine model, administration of niacin (30 mg/kg intraperitoneally) increased serum adiponectin, an insulin sensitizer, and reduced lipolysis in wild-type mice 60 min after injection but not in GPR109A-deficient mice [47]. Levels of adiponectin are positively correlated with high-density lipoprotein (HDL) cholesterol levels, potentially due to HDL-induced adiponectin gene expression [48]. In a retrospective analysis of 34 patients with stage 2–4 CKD (estimated glomerular filtration rate (eGFR) 15 to 89 mL/min/1.73 m^2^), niacin 500 mg/day for 6 months reduced serum levels of triglycerides and phosphate, while increasing plasma HDL cholesterol levels and eGFR (11). Niacin may therefore regulate insulin homeostasis through the assembly of distinct HDL cholesterol particles but this is a hypothesis to be tested.

In healthy individuals, optimal insulin levels are maintained by pancreatic islet beta-cell production and secretion and by insulin removal from plasma by liver (50–70%) and kidney (30–50%) [49]. In CKD, insulin resistance is common, even in the absence of diabetes [49]. Mechanisms that contribute to insulin resistance in CKD (e.g., systemic inflammation, oxidative stress, metabolic acidosis) [21] are also associated with hypoxia. Hypoxia-induced cell signals in adipocytes promote lipolysis but suppress insulin signaling through reduced insulin receptor activation and glucose transporter 4 (GLUT4) production in human and murine adipocytes [50]. These data highlight the complexity of responses involving insulin receptors, HCARs, HIF-1α, and AHR (Fig. 5).

Moreover, HCAR2 is also expressed by murine renal tubular epithelial cells in the context of sepsis-associated acute kidney injury (AKI) [51]. Treatment with lactate (lactated Ringer's solution) attenuated AKI severity and reduced mortality in wild-type but not HCAR2 deficient mice [51]. Thus, the benefit of lactate-induced HCAR1 appears to depend on HCAR2 activation and its downstream effects. A more complete understanding of these separate but interdependent pathways may lead to better therapeutics for insulin resistance in the presence or absence of CKD, and perhaps a better understanding of AKI pathogenesis.

### The niacin non-conjugative pathway and injury

Niacin contributes to NAD biosynthesis through the non-conjugative (Preiss-Handler) pathway [52]. Niacin is transported across plasma membranes by monocarboxylate transporters (SLC6A8/SMCT1, SLC5A12/SMCT2 and SLC16A1/MCT1). Intravenous administration of radionuclide-labeled [^11^C]niacin to mice revealed that the highest tissue uptake occurs in the kidneys, which is consistent with high *Smct* mRNA expression levels in the renal cortex and outer medulla [53]. This study also showed higher levels of niacin in male compared to female mice.

Inside the cell, niacin is transformed by the rate-limiting enzyme of this pathway, nicotinic acid phosphoribosyltransferase (NAPRT), to nicotinic acid mononucleotide, prior to the generation of NAD (Fig. 3). NAPRT is not only essential for NAD production but also contributes to the prevention of oxidative stress associated with hypoxia and cellular injury [54]. NAPRT can be released into the extracellular microenvironment (eNAPRT) and serves as a damage-associated molecular pattern (DAMP), activating toll-like receptor (TLR)-4, as occurs in patients with sepsis [54]. The function of eNAPRT in CKD has not been fully explored. However, in mice subjected to 5/6 nephrectomy and continuous low-dose angiotensin II infusion, wild-type mice, but not TLR4 mutant mice, develop progressive CKD [55], suggesting that a TLR4 ligand, such as eNAPRT, could contribute to progressive renal disease.

### The NAD salvage pathway

NAD is predominantly synthesized through the salvage pathway. NAM is produced as a by-product of the actions of NAD in modifying protein substrates [e.g., sirtuins and poly-ADP ribose polymerase (PARPs)] and serves as the primary metabolic precursor. The enzyme NAMPT catalyzes the transformation of NAM into nicotinamide mononucleotide (NMN). Outside the cell, CD73 (an ecto-5ʹ-nucleotidase) catalyzes the transformation of NMN into nicotinamide riboside (NR) and NR is internalized through the equilibrative nucleoside transporter 1 (ENT1), encoded by *SLC29A1,* which acts to achieve similar levels of the transported molecule on both sides of the plasma membrane, whereas a concentrative transporter promotes cell entry over cell exit of a particular molecule. Inside the cell NR is transformed to NMN through an NR kinase [56]. NMN produced by either an NR kinase or NAMPT is transformed to NAD through nicotinamide mononucleotide adenylyltransferases (NMNAT1-3) and an NAD synthetase (NADSYN1) [57, 58] (Fig. 3).

### The protective role of NAM in CKD

In experimental CKD, NAM has primarily been tested as a preventive therapy. In mice fed an adenine-rich diet to induce tubulointerstitial and at the same time provided with NAM-supplemented drinking water over six weeks, NAM supplementation reduced kidney fibrosis and inflammation compared to controls. However, when mice were fed an adenine-rich diet for six weeks, NAM administered for the following four weeks did not reduce renal fibrosis [31]. In two studies using the unilateral ureteral obstruction (UUO) model, daily administration of intraperitoneal NAM, commencing either one hour or three days before the operation and continuing through at least seven days post-operation, inhibited progressive tubulointerstitial fibrosis, inflammation and renal injury [29, 30]. These studies therefore highlight the prophylactic effects of NAM on kidney damage following kidney injury.

In vitro, NAM reduces both HIF-1α stability and HIF-1α-induced activation of human macrophages, acting in part through increased SIRT1 activation [25]. In mice with tubular cell-specific HIF-1α-overexpression, increased acetylated-HIF-1α induced apoptosis and tissue fibrosis [59], suggesting that SIRT1 activity could modulate the response. NAM (0.25 mg/g/day intraperitoneally administered 3 days before UUO) induced SIRT1 activation in vivo and reduced acetylation of SMAD3, p53, and forkead-box (FOX)-O1 proteins in kidney cortices [29]. Interventions that decrease the activity of either Smad3 [60] or p53 [61] also ameliorate disease in animal CKD models. Smad3 and p53 activation are inhibited by deacetylation, whereas FOXO1 is activated by deacetylation [60].

Dysregulation of FOXO1 is associated with diabetic kidney disease, encouraging investigational research of FOXO1 as a therapeutic target [62]. Acetylated FOXO1 inhibits its DNA binding affinity and promotes FOXO1 phosphorylation and degradation in the cytoplasm [63]. In a murine model of endotoxin-induced AKI, Foxo1 and peroxisome proliferator-activated receptor-gamma coactivator (Pgc)-1α (a possible downstream target of Foxo1) are reduced in proximal tubular epithelial cells at the mRNA and protein levels [64]. PGC-1α, like FOXO1, is deacetylated by SIRT1, which promotes its activation and function in mitochondrial biogenesis [65] and in the production of quinolate phosphoribosyl transferase (QPRT) in the de novo pathway [27]. Intrarenal adeno-associated virus delivery of a viral vector carrying the *Foxo1* gene in murine endotoxin-induced AKI demonstrated that increased Foxo1 protein levels were associated with improved renal function [64], suggesting a protective function of FOXO1 activation in CKD.

Alternatively, in an I/R model, Foxo1 protein levels increased and phosphorylated-Foxo1 levels decreased, suggesting that FOXO1 activation may be a pathogenic component driving kidney disease [66]. Intraperitoneal injections of a Foxo1 inhibitor (AS1842856) in this model reduced kidney injury, improved mouse survival and kidney function, and abrogated I/R-induced inhibition of Pgc-1α through increased activation of the Pgc-1α transcription factor, cAMP-response element binding protein (Creb).

**Fig. 6:**
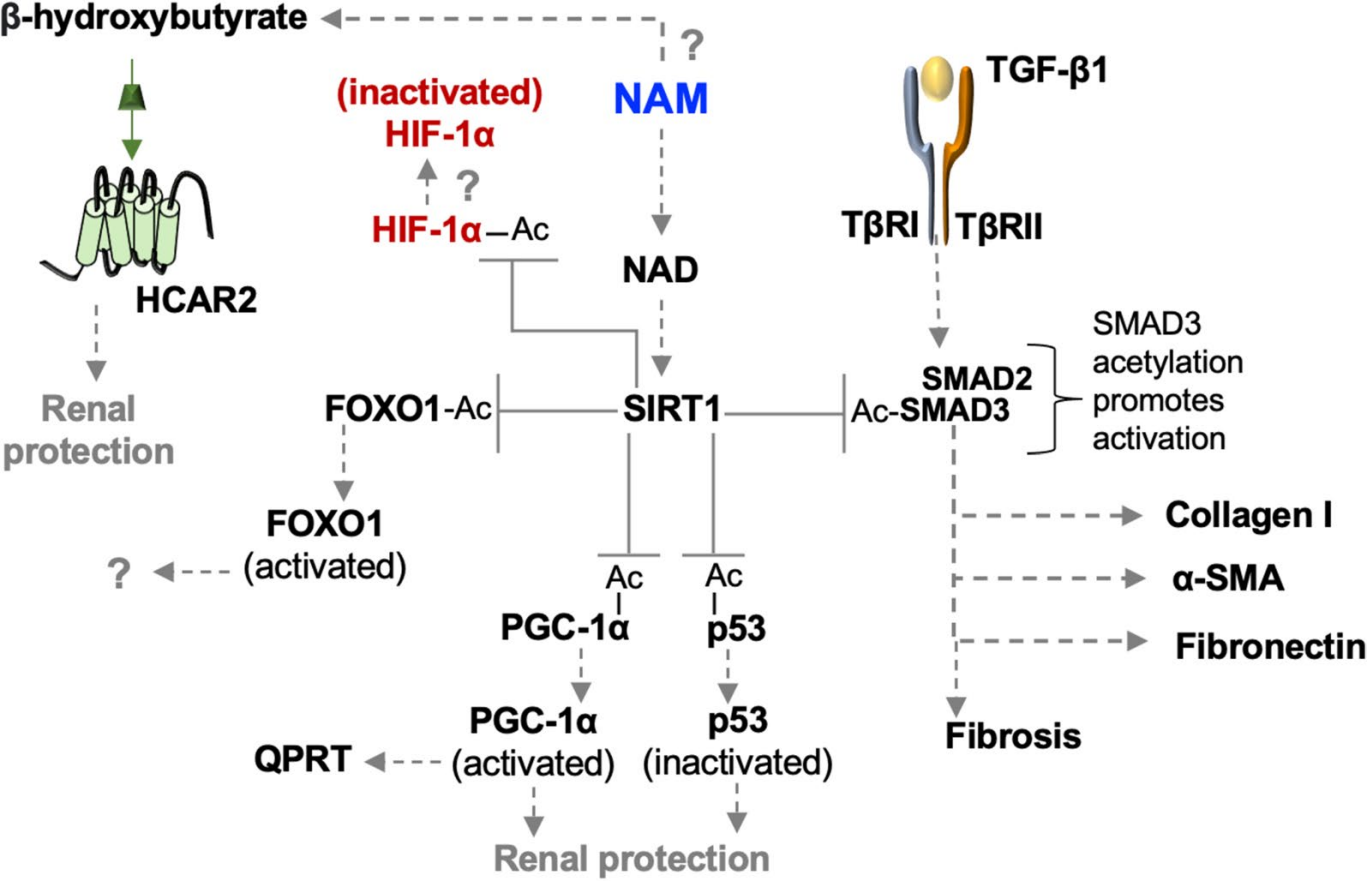
**NAM function in CKD**. NAM induces the activity of SIRT1 and promotes the deacetylation of SMAD3, p53, PGC-1α, and FOXO1. Deacetylation of SMAD3 inhibits fibrosis. Deacetylation of p53 inhibits apoptotic injury. Deacetylation of PGC-1α promotes its activation, which is associated with mitochondrial biogenesis and the production of the de novo pathway enzyme, quinolinate phosphoribosyl transferase (QPRT). Deacetylation of FOXO1 promotes its activation. The functions of FOXO1 in CKD are not fully explored. PGC-1α levels decrease in CKD. NAM-induced production of β-hydroxybutyrate and activation of HCAR2 may improve kidney function under conditions of PGC-1α deficiency, acting through unknown downstream mechanisms. SIRT1 may affect the activity of HIF-1α

Pgc-1α expression falls during experimental renal ischemia. Renal function also worsens in Pgc-1α deficient mice in association with lower renal levels of NAM. Intraperitoneal NAM (400 mg/kg/day), administered four days prior to I/R injury in Pgc-1α deficient mice, prevented post-ischemic AKI and also normalized post-ischemic fat accumulation [67]. In this study, NAM induced β-hydroxybutyrate activation of HCAR2, and intraperitoneal injections of an HCAR2 inhibitor (mepenzolate bromide) abrogated renal protection. Thus, niacin directly whereas NAM indirectly regulates HCAR responses (Figs. 5 and 6).

In summary, FOXO1, PGC-1α, SMAD3, and p53 are all important in mediating the effects of NAM in experimental CKD. Each of these proteins may be modulated by NAM-induced SIRT1 activity. NAM downstream responses and mechanisms may include increased mitochondrial biogenesis (e.g., PGC-1α activation), production of antioxidants (e.g., FOXO1-induced catalase), reduced fibrosis (SMAD-3-induced α-SMA), and renal protection (e.g., p53 inactivation). Further exploration of these mediators of NAM action may prove beneficial to understanding the potential role of NAM in the treatment of kidney disease (Fig. 6).

### CKD-induced changes in the NAM methionine cycle

The synthesis of MNA is carried out by nicotinamide N-methyltransferase (NNMT), which catalyzes the transfer of a methyl group from S-adenosylmethionine (SAM) to NAM, producing S-adenosylhomocysteine (SAH) and methyl nicotinamide (MNA). Subsequently, MNA is metabolized to n-methyl-2-pyridone-5-carboxamide (N-Me-2PY) and n-methyl-4-pyridone-3-carboxamide (N-Me-4PY) via an aldehyde oxidase (AOX) [68]. The human genome contains one AOX gene (*AOX1*), whereas the mouse genome encodes four Aox genes (*Aox1, Aox2, Aox3, Aox4*) [69]. Immunostains of human tissue identified expression of AOX1 protein in various human tissues, including the adrenal gland and kidney (with expression in proximal, distal, and collecting tubules but not in glomeruli) [70]. AOX enzymes are an important source of reactive oxygen species (ROS) [69], which can potentiate tissue injury and hypoxia.

In a small 1956 dietary study of healthy males (eight subjects per group), urinary MNA excretion was assessed [71]. At baseline, MNA excretion was 3.64–4.80 mg/day. Subjects were given a basal diet or diets supplemented with 2 mg riboflavin, alone or in combination with either 50 mg L-tryptophan or 10 mg nicotinamide. After nine weeks, urinary excretion of MNA decreased for subjects on diets that were non-supplemented (0.96–2.37 mg/day), or supplemented with riboflavin (0.59–2.54 mg/day) and L-tryptophan (1.78–3.43 mg/day) but MNA excretion increased in subjects on the nicotinamide-supplemented diet (3.08–6.56 mg/day). Comparing values at 16, 24, 36 and 46 weeks on experimental diets, the authors concluded that NAM supplementation caused higher efficiency in MNA excretion compared to L-tryptophan at a factor of 1:60. These data would suggest that higher levels of MNA, N-Me-2PY and N-Me-4PY determined in CKD patients are sourced from the NAD salvage or Preiss-Handler pathways (Fig. 3).

In a cross-sectional study of 139 patients with CKD, serum levels of NAM and NMN decreased, while levels of N-Me-2PY and N-Me-4PY increased with progressive renal dysfunction [72]. Moreover, in a murine model of UUO, kidney tissue levels of NAM, NMN, and NAD decreased, while MNA, N-Me-2PY, and N-Me-4PY levels accumulated in the obstructed kidneys, compared to the sham-operated kidneys [72]. This model also demonstrated that renal fibrosis was reduced in Nnmt-deficient mice, possibly as a result of reduced levels of Aox-induced uremic toxins in the model (Fig. 3).

In another murine study using the UUO model, NNMT was also upregulated in the kidney, and Nnmt protein levels correlated with renal fibrosis [73]. Despite increased NNMT levels in kidney tissue, hydrodynamic-based injections of *Nnmt* plasmids two days before and after surgery were administered to induce NNMT overexpression. Either NNMT over-expression or MNA gavage administration ameliorated renal fibrosis, possibly as a result of transforming growth factor (TGF)-β inhibition [73]. The benefits of *Nnmt*-expressing plasmids may also be associated with MNA production and its downstream anti-inflammatory [74], anti-thrombotic [75], and vasorelaxing [76] properties. Because the absence of *Nnmt* and the injections of *Nnmt*-expressing plasmids each provided benefits, further study of NNMT and its downstream metabolites (MNA, N-Me-2PY, and N-Me-4PY) in tissue, blood, and urine in human CKD may be warranted (Fig. 4).

Interestingly, reduced urinary MNA levels may be a biomarker of renal functional decline and of mortality. In assessing urinary MNA prospectively in 660 kidney renal transplant recipients compared to 275 healthy kidney donors, renal transplant recipients excreted less urinary MNA compared to healthy donors; this was associated with higher premature all-cause mortality, independent of other predictors [77]. However, in assessing the urine metabolites of 82 US military personnel admitted to an ICU with traumatic injuries, elevated urinary MNA and lactate were associated with higher AKI stage, increased mortality, and need for renal replacement therapy [78]. Differences between these studies may depend upon the activity of NNMT and AOX enzymes and the levels of urinary N-Me-2PY and N-Me-4PY. A possible function of AOX1 as a biomarker in CKD has not been fully explored [68].

### Increased extracellular NAMPT is a CKD biomarker

NAMPT is the rate-limiting enzyme in the NAD salvage biosynthesis pathway. Like erythropoietin [14], NAMPT is primarily regulated by HIF-2α, which is stabilized by SIRT2 activity and therefore by NAD [17, 79]. NAMPT has functions both outside and inside the cell. Extracellular NAMPT (eNAMPT) is a pro-inflammatory cytokine, commonly released from B lymphocytes (also known as pre-B cell colony-enhancing factor/PBEF) and from adipocytes (also known as visfatin), indicating a hypothesized role in visceral fat biology [54]. Like eNAPRT, eNAMPT, can also activate TLR4 [80] and potentially affect TLR4 responses in CKD [55] (Fig. 3).

In patients with diabetes, serum eNAMPT levels are elevated and are positively associated with insulin resistance and higher urinary albumin-to-creatinine ratios [81]. Plasma eNAMPT levels are also increased in non-diabetic, non-proteinuric hypertensive outpatients and associated with eGFR decline [82]. In CKD patients, plasma eNAMPT levels are an indicator of disease severity and a marker of endothelial damage [83]. These findings may reflect the release of eNAMPT from adipocytes and kidney cells, particularly since decreased intracellular NAMPT and sirtuin 6 staining are found in the proximal tubules of diabetic patient kidneys manifesting severe fibrosis compared to healthy controls [84]. In an adenine-induced CKD mouse model [31] and in Zucker diabetic obese rats [85], decreased intracellular NAMPT levels may reduce levels of NAD in the kidney. Understanding the mechanisms contributing to the cellular release of NAMPT and its dual role as an enzyme in extracellular NAD metabolism versus ligand activation of TLR4 may lead to novel therapeutics for CKD.

### The protective role of NMN in CKD

NAMPT catalyzes the formation of NMN. In mice placed in a hypoxic chamber (10.8% O_2_) for four weeks, NMN (500 mg/kg administered intraperitoneally, every third day) attenuated HIF-1α activation-induced adipose fibrosis and inflammation [86]. In an I/R model, NMN (500 mg/kg intraperitoneally) administered 20 min before I/R and daily for 3 days after the procedure or on day 3 and 14 after I/R, NMN also reduced inflammation (expression of IL-6, IL-8, TGF-β1) and fibrosis (deposition of collagen IV, α-SMA) [87].

**Fig. 7:**
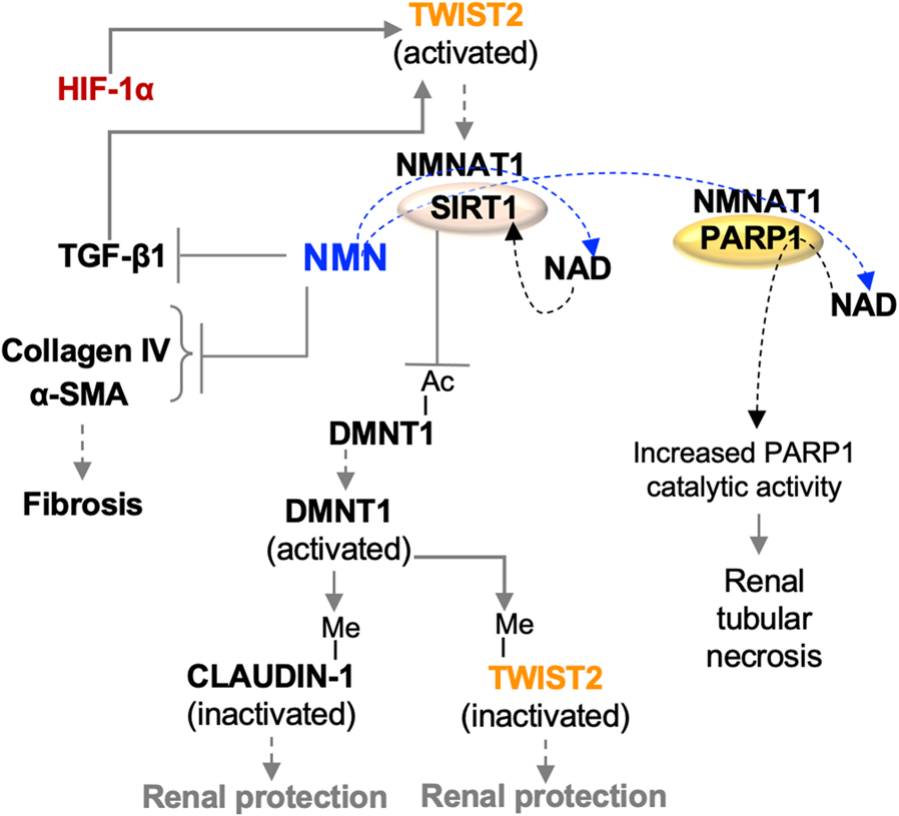
**NMN function in CKD**. NMN inhibits the functions of TGF-β1 and other factors involved in fibrosis. HIF-1α and TGF-β1 cell signals promote the activation of Twist2, which in turn activates nicotinamide mononucleotide adenylyltransferases (NMNAT1). NMNAT1 catalyzes the transformation of NMN into NAD. Poly-ADP ribose polymerase (PARP1) and SIRT1 can recruit NMNAT to promote their catalytic activity. PARP1 activity is associated with renal tubular necrosis. SIRT1 activity. SIRT1 deacetylates DNA methyltransferase (DNMT1) and promotes its activation. DNMT1 methylates CLAUDIN-1 and TWIST2 and is renal protective

In mice given intraperitoneally Adriamycin (doxorubicin) to induce focal segmental glomerulosclerosis (FSGS), NMN (500 mg/kg intraperitoneally) administered daily for 14 days improved kidney function and reduced serum cholesterol levels compared to controls [88]. The NMN-driven mechanisms in this model include increased renal tissue levels of SIRT1 and NAMPT but reduced levels of PARP1 and NMNAT1. The model also supports NMN-induced SIRT1 deacetylation and activation of DNA methyltransferase (Dnmt1), which subsequently methylates and reduces levels of claudin-1, a molecule associated with podocyte effacement [89] (Fig. 7).

Expression and production of the transcription factor twist family basic helix-loop-helix transcription factor 2 (Twist2) is increased in kidney tissue in a mercuric chloride rat model of AKI [90]. Twist2 can be activated by a variety of cell signals, including those promoted by HIF-1α and TGF-β1 [91]. In the Adriamycin (doxorubicin)-induced mouse model of FSGS [88], NMN decreased Twist2 activity through Dmnt1-induced methylation, which in turn reduced Twist2-induced *Nmnat1* gene expression. PARP1 recruits NMNAT1 to promoters (e.g., *SOCS2,* encoding the suppressor of cytokine signaling) and there NMNAT1 produces NAD to support the catalytic activity of PARP1 and its PARylation of proteins acting on these gene promoters [92]. PARP1 expression is increased in the proximal tubule and renal vasculature and therapeutically targeted in CKD [23]. Thus, NMN participates in a regulatory loop controlled indirectly by SIRT1 (Fig. 7). Like PARP1, SIRT1 recruits NMNAT1 to regulate the deacetylation of proteins at promoter sites [93]. Further study into the recruitment of NMNAT to SIRT1 versus PARP1 may provide additional therapeutic strategies to slow CKD progression by using NMN.

### The protective role of NR in CKD

Extracellular NMN is transformed to NR by the ectoenzyme CD73 (an immune checkpoint molecule) and transported into the cell through ENT1 [56] (Fig. 3). In a rat I/R model of AKI, reduced kidney tissue NAD and SIRT1 levels were restored with NR (500 kg/mg/day) by gavage administration for two weeks prior to surgery and one day after surgery [94]. However, injury (e.g., higher expression levels of neutrophil gelatinase-associated lipocalin) and fibrosis (e.g., reduced protein levels of α-Klotho) in the model was not affected by NR prophylactic treatment.

Another study examined three standard models of kidney injury (I/R, cisplatin-induced AKI, and UUO) and the podocyte-apoptosis through targeted activation of caspase-8 (POD-ATTAC) murine model. The latter contains an inducible FK-506 binding protein (FKBP)-caspase 8 fusion transgene in the podocin promoter, causing podocyte apoptosis in response to intraperitoneal injections of the dimerizer molecule AP20187 [95]. In mice given chow supplemented with NR (400 mg/kg/day) for 10 days and subjected to ischemia/reperfusion or cisplatin AKI, tubular injury decreased and kidney function improved compared to controls at the 48 h timepoint. However, mice pretreated with chow containing 800 mg/kg/day NR for seven days and subjected to UUO or POD-ATTAC for 14 days did not manifest improved kidney function or reduced fibrosis. Thus, this study did not support oral administration of NR as an approach to slow CKD in mice.

**Fig. 8:**
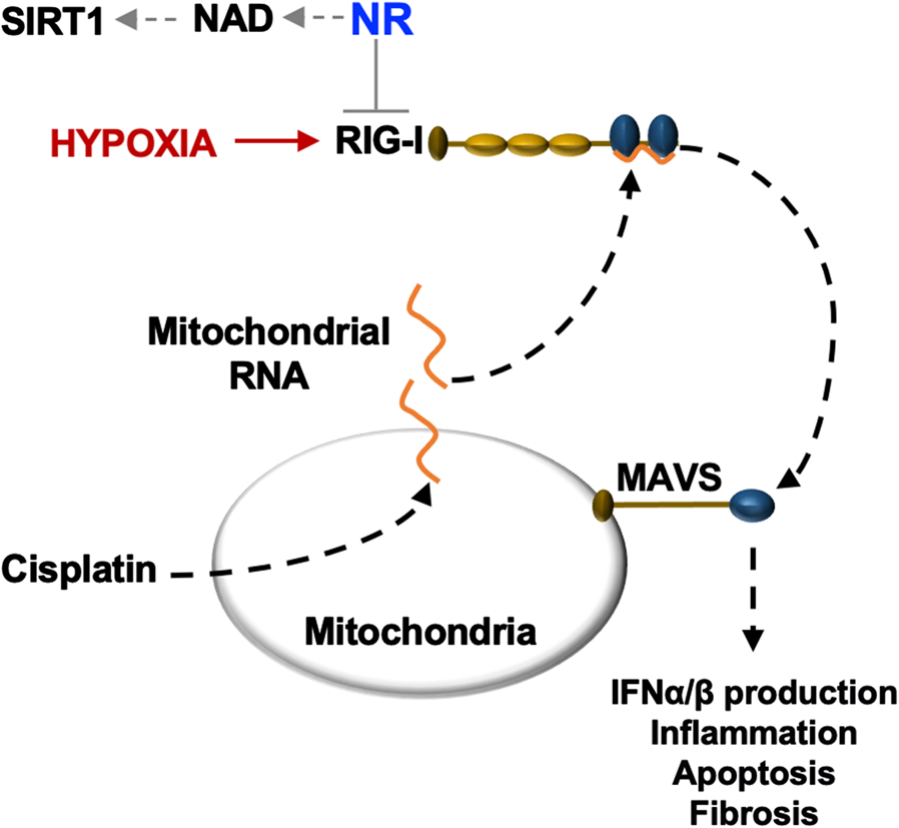
**NR function in CKD**. Mitochondrial damage can occur in cisplatin-induced kidney injury models, causing the release of mitochondrial RNA. Immunostimulatory RNA, such as mitochondrial RNA, causes the activation of the cytosolic pattern recognition receptor retinoic acid-inducible gene I (RIG-I). Conformational changes in activated RIG-I facilitates RIG-I binding to mitochondrial antiviral-signaling protein (MAVS) and subsequent signals involved in renal injury. Hypoxia induces the production of RIG-I and NR antagonizes RIG-I. NR may mediate its effects via increased NAD and SIRT1 activity

In examining the effect of NR (435 mg/kg) or NMN (500 mg/kg) injected intraperitoneally for four consecutive days, commencing two hours before I/R or cisplatin-induced AKI, either NR or NMN normalized kidney tissue NAD levels, reduced kidney injury and improved kidney function compared to controls [96]. This study also showed that cisplatin exposure causes cytosolic leakage of mitochondrial RNA and consequent activation of the cytosolic pattern recognition receptor retinoic acid-inducible gene-I (*RIG-I*). Subsequent use of RIG-I knockout mice in the study reduced cisplatin-induced kidney injury that was not further improved by NR supplementation, suggesting that RIG-1 is an NR target. RIG-I is increased in cultured NRK52E rat kidney epithelial cells exposed to hypoxia and in murine kidneys subjected to renal artery clamping for 10 min [97]. These observations may support a role for NR-induced NAD in regulating hypoxia-induced RIG-I expression (Fig. 8).

### The NAD de novo pathway

In the NAD de novo pathway, three enzymes regulate the catabolism of tryptophan: indoleamine-2,3-dioxygenase (IDO)1, IDO2, and tryptophan dioxygenase (TDO). Of these three enzymes, IDO1 is the rate-limiting step in catalyzing the oxidative cleavage of tryptophan into n-formyl-l-kynurenine due to the lower substrate specificity of IDO2 and TDO [98]. n-formyl-l-kynurenine is rapidly converted to l-kynurenine by a formamidase. In the production of NAD, a kynurenine 3-monooxygenase converts l-kynurenine to 3- hydroxy-l-kynurenine, kynureninase converts 3- hydroxy-l-kynurenine to 3-hydroxy-anthranilic acid (HAA) and HAA dioxygenase converts 3-HAA to quinolinic acid. In the final steps QPRT transforms quinolinic acid to the common Preiss-Handler pathway product, nicotinic acid mononucleotide (NAMN), which is converted to NAD through NMNAT1-3 and an NAD synthetase [58] (Fig. 3).

### Elevated kynurenine/tryptophan ratio (IDO activity) is a CKD biomarker

The kynurenine/tryptophan ratio in blood or urine has been termed IDO activity. IDO activity in blood [22, 99, 100] or urine [101] is increased in CKD patients compared to healthy controls and is an indicator of disease severity. In IDO1 deficient mice, serum levels of kynurenine are similar compared to wild-type mice, but are lower in mice who received for two weeks a diet supplemented with 0.25% adenine, compared to controls, suggesting that CKD primarily requires IDO1 over IDO2 and TDO [102].

IDO1 protein levels are increased in cortical and medullary tubules, and are associated with fibrosis in models of UUO [103, 104] and I/R [105, 106]. These fibrotic responses may involve increased activation of the Wnt/β-catenin pathway. Specifically, in I/R AKI involving *Ido1*-deficient mice, whole kidney homogenates revealed lower immunoblot levels of fibrotic markers (e.g., α-SMA, fibronectin, and vimentin) and the Wnt/β-catenin pathway markers (e.g., glycogen synthase kinase-3 (GSK-3β) and β-catenin) compared to controls [105]. This study also indicated that I/R AKI mice treated with prostaglandin E2 (PGE2, 0.5 mg/mL administered daily interperitoneally for 14 days) generated similar results to the IDO1 deficient mice. PGE2 is also known to induce the transcription and production of IDO1 [107], highlighting the complexity of this response that may depend on the select activation of one of the four PGE2 receptors [108].

Two particular IDO1 inhibitors are a tryptophan mimetic, 1-methyl-D-tryptophan (1-MT), and a suicide inhibitor, (e.g., linrodostat), which binds to the enzyme in a chemically unreactive state and once bound, linrodostat irreversibly inactivates the enzyme [109]. In UUO mice, IDO1 protein levels were elevated and treatment with 10 mg/mL of either 1-MT or linrodostat intraperitoneally, twice daily starting at day zero through day seven, reduced UUO-induced collagen deposition [104]. To understand the mechanisms responsible for the response, TGF-β-treated murine precision-cut kidney slices were treated with these inhibitors, revealing decreased expression of collagen type 1, fibronectin, and α-SMA. In an I/R AKI model, mice treated with 1-MT (3 mg/mL, twice daily) 1 h pre- and 48 h post-ischemia ameliorated kidney function [106]. Mice pretreated with 1-MT (50 mg/kg, twice daily for two days) and fed the 0.25% adenine supplemented diet 14 days to induce CKD showed reduced levels of blood kynurenine, tissue factor expression in the arteries, and thrombophilia compared with respective controls [102].

In summary, increased IDO activity promotes catabolism of tryptophan and production of kynurenine, fibrosis, injury, and thrombosis associated with kidney injury. Further exploration of the kynurenine receptor, AHR [20], may identify additional downstream targets associated with IDO activity.

### Kynurenic acid versus 3-hydroxy-l-kynurenine production in CKD

Kynurenine is metabolized by kynurenine 3-monooxygenase (KMO) in the NAD biosynthesis de novo pathway to 3-hydroxy-L-kynurenine (3HK) or transformed by kynurenine aminotransferases (KAT) to generate kynurenic acid, which binds HCAR3, G-protein coupled receptor 35 (GPR35) and AHR [110] (Figs. 3 and 5). In patients with autosomal dominant polycystic kidney disease, greater CKD severity was associated with increased plasma IDO activity and kynurenic acid levels [111], suggesting that increased KAT transaminase activity promotes disease progression.

However, in an I/R model of AKI, KAT transaminase activity demonstrated a benefit. Specifically, mice deficient in KMO activity showed improved kidney function, reduced tubular epithelial apoptosis, and decreased kidney neutrophil infiltration associated with reduced plasma levels of 3HK and higher levels of kynurenine and kynurenic acid compared to controls [112]. These observations highlight a distinct KAT/kynurenic acid function in KMO deficient mice.

**Fig. 9:**
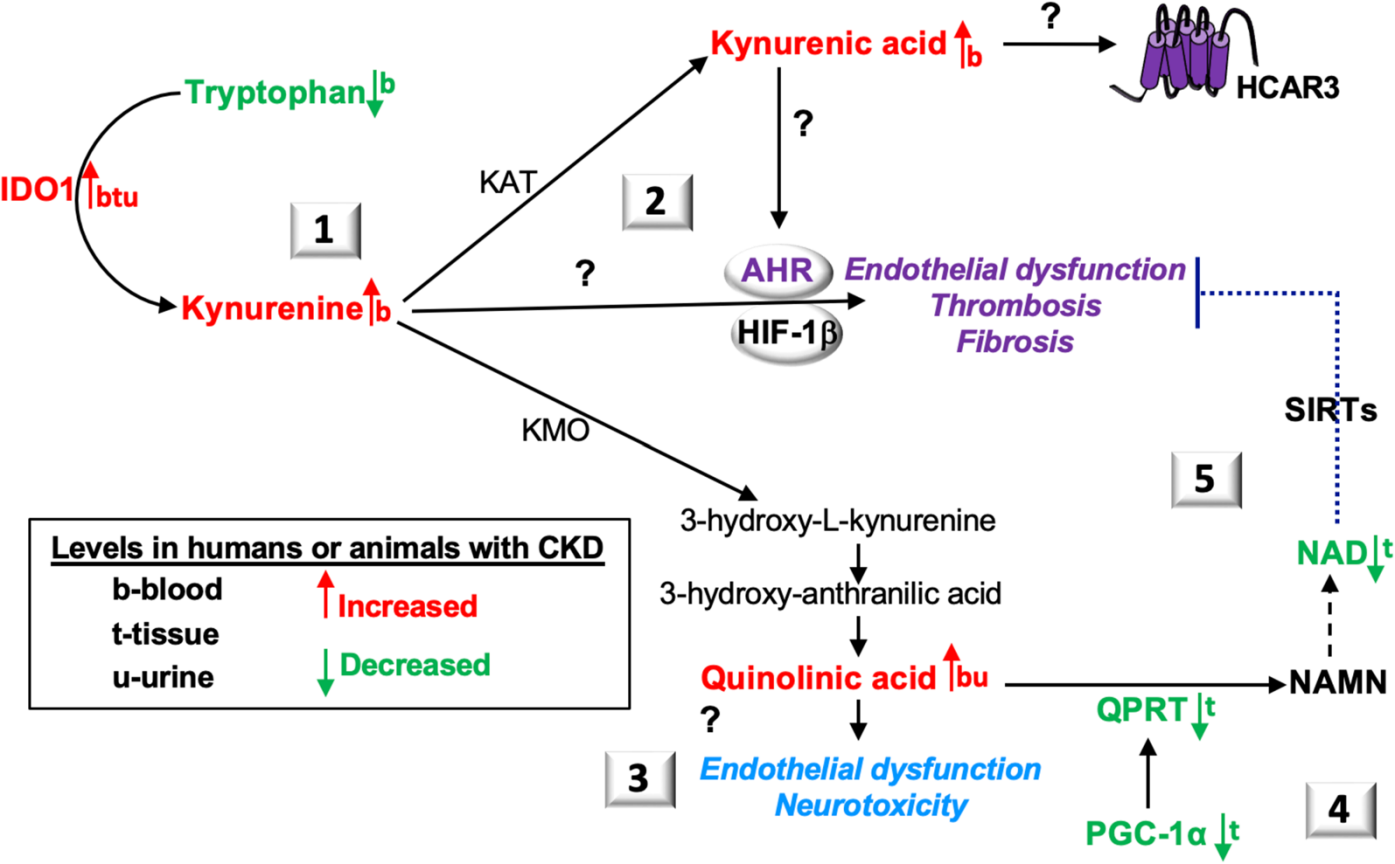
**The de novo pathway in CKD**. (1) Elevated indoleamine 2,3-dioxygenase 1 (IDO1) activity in CKD increases the transformation of tryptophan into kynurenine. Kynurenine aminotransferases (KAT) and kynurenine 3-monoxygenase (KMO) transform kynurenine into kynurenic acid and 3-hydroxy-l-kynurenine, respectively. (2) Both kynurenine and kynurenic acid bind and activate the aryl hydrocarbon receptor (AHR). AHR transcribes genes with its dimer partner, hypoxia inducible factor (HIF)-1β. AHR ligands are associated with endothelial dysfunction and thrombosis. Kynurenic acid also binds and activates hydroxy-carboxylic acid receptor (HCAR)-3. The binding affinity of these ligands for AHR or HCAR3 and their downstream responses have not been fully explored in CKD. (3) Quinolinic acid (also known as quinolinate) is upregulated in CKD. Higher quinolinic acid levels can be correlated with endothelial dysfunction. Quinolinic acid is also a neurotoxin. Quinolinic acid functions in CKD have not been fully explored. (4) Downregulation of peroxisome proliferator-activated receptor-gamma coactivator (PGC)-1 α, which transcribes quinolinate phosphoribosyl transferase (QPRT), prevents the conversion of quinolinic acid into nicotinic acid mononucleotide (NAMN). (5) NAD levels are reduced in CKD. NAD-induced activation of SIRT1 antagonizes the activity of AHR and its downstream responses. Enzymes and molecules altered in CKD are highlighted (see text for additional details)

In summary, kynurenine is catabolized by KMO or KATs into 3HK or kynurenic acid, respectively. Whether kynurenine or kynurenic acid exhibit different binding affinities for AHR or produce differential downstream responses has not been fully examined (Fig. 9). In CKD, both ligands are elevated (Table 1). Further investigation of the divergent pathways involving kynurenic acid and 3HK may offer insight into the production of AHR ligands in CKD (Table 1).

**Table 1:** Tryptophan metabolites that can bind AHR and are expressed in CKD

AHR ligand (possible function)	AHR ligand reference levels in CKD	Enzymatic source of ligand formation
Indoxyl sulfate [24] (activates endothelial oxidative stress/endothelial dysfunction)	Elevated in serum, 23.1 mg/mL versus 0.53 mg/mL in healthy subjects [152]	Gut bacteria
Indole-3-acetic acid [123] (inhibits endothelial cell angiogenesis)	Elevated in serum, 2 mg/mL versus 0.5 mg/mL in healthy subjects [152]	Gut bacteria
Indole 3-propionic acid [153] (activates macrophage phagocytosis)	Reduced in serum, 34.7 ng/mL versus 49.8 ng/mL in healthy subjects [154]	Gut bacteria
Kynurenic acid [155] (induces epithelial IL-6 production, also an HCAR3 ligand)	Elevated in serum, 151.0 ug/L versus 5.48 ug/mL in healthy subjects [152]	Kynurenine aminotransferases
Kynurenine [156] (activates T regulatory cells and immunosuppressive myeloid cells)	Elevated in plasma (uM), CKD stage 3 (2.61), CKD stage 4 (3.17), CKD stage 5 (3.72) versus health subjects (1.84) [157]	IDO1

### Elevated quinolinate/tryptophan ratio is a CKD biomarker

In an unbiased metabolomics screen of urine from mice following I/R AKI, quinolinate (also known as quinolinic acid) was identified as one of the 27 metabolites with a two-fold increase out of 204 examined [27]. Assessing QPRT in the study showed that the protein was abundant in the wild-type proximal tubule epithelium and reduced at the mRNA level in the I/R kidney. In humans, metabolic profiling of urine samples collected at baseline and the day after cardiopulmonary bypass in 41 cardiac surgery patients showed that the quinolinate/tryptophan ratio was higher one day after surgery in the 11 patients that later developed AKI [113]. In plasma from patients with end-stage kidney disease, the quinolinate/tryptophan ratio also increased compared to healthy controls [114]. The values also trended higher in certain conditions (patients on maintenance hemodialysis > patients on continuous ambulatory peritoneal dialysis > chronic renal failure) and were correlated with factors (thrombomodulin and von Willebrand factor) of endothelial dysfunction. Thus, quinolinate relative to tryptophan in urine or plasma may be a biomarker of CKD.

Moreover, examination of the human kidney-2 (HK-2) cell line indicated that lower QPRT activity may be associated with increased endoplasmic reticulum stress (ER) responses [114]. In human myotubes and mouse skeletal muscle, palmitate-induced ER stress down-regulates PGC-1α production [115]. In a murine I/R AKI model, overexpression of Pgc-1α inhibited ER stress and improved kidney function [116]. QPRT is, in part, regulated by PGC-1α [27], which declines in experimental renal ischemia [67]. Because quinolinate is also a neurotoxin [117], understanding QPRT regulatory networks may provide further insight into NAD depletion and neurological complications that can develop in CKD [118] (Fig. 9).

### AHR in NAD metabolism

AHR ligands are elevated in CKD and produce endothelial dysfunction and immune suppression (Table 1). AHR and HIFs dimerize with HIF-1β to transduce cell signals. PARP7 (2,3,7,8-tetrachlorodibenzo-p-dioxin (TCDD)-inducible poly(ADP-ribose) polymeras, TiPARP) is a downstream target of AHR that co-localizes with either HIF-1α or AHR in the recruitment of an E3 ligase for ubiquitin-mediated proteasome degradation of either transcription factor [20] (Fig. 1). In chick embryos treated in ovo with the potent AHR ligand, dioxin (2,3,7,8-tetrachlorodibenzo-p-dioxin), PARP7 activity increased and NAD levels reduced in the thymus and liver [119]. Administering NAM, NR, or the pan-PARP inhibitor PJ34 in this model increased cellular NAD levels. In another study of chick embryo livers, NAM treatment reversed dioxin-induced NAD depletion, *PARP7* expression, PGC-1α protein acetylation (inactivation), and PGC-1α production [120]. In this study, activated AHR also reduced SIRT1 levels, suggesting that AHR may reduce PGC-1α activity by reducing SIRT1 deacetylation of PGC-1α [121]. Thus, AHR may be an additional factor in NAM-induced responses (Fig. 6).

Stimulation of human umbilical vein endothelial cells (HUVECs) with uremic toxins, indoxyl sulfate [122] or indole-3-acetic acid [123], induces the production of reactive oxygen species and inhibits angiogenesis, respectively. Indoxyl sulfate stimulated HUVECs also increase gene expression of *PARP7* and *F3* (tissue factor). Adding the heat-shock 90 protein inhibitor, geldanamycin, which also inhibits AHR activity, or AHR siRNA, to the indoxyl sulfate stimulated cells decreased tissue factor expression and production [124]. Similar responses were observed with the uremic toxin, indole-3 acetic acid, which was subsequently revealed to up-regulate tissue factor in endothelial cells through AHR-regulated NF-κB activation [125]. In various studies, NAM inhibited NF-κB through decreased phosphorylation and subsequent degradation of its inhibitor of NF-κB [126], reduced production of the p65 subunit [25], and reduced the acetylation (activation) the p65 subunit [25, 127]. Thus, NAM may also affect AHR by regulating NF-κB activity. Further exploration of the NAM response in AHR cell signals may provide insight into the pathology of the receptor in CKD [20].

Lastly, in analyzing human plasma from patients with CKD, circulating tissue factor levels were elevated and correlated with increased levels of indoxyl sulfate and indole-3 acetic acid compared to levels in healthy subjects [124]. In vitro, NAM inhibits tissue factor produced by human pancreatic islets isolated from human cadaver donors [128]. NAM also inhibits cell surface expression of tissue factor induced by endotoxin on human monocytes [129]. Possibly, NAM affects circulating tissue factor levels in patients with CKD.

### Future perspectives

As reviewed here, NAD metabolism is dysregulated in CKD. Identified biomarkers include NAD metabolites and enzymes from the NAD de novo and salvage pathways released into blood and urine. Mechanisms that control increased production (e.g., IDO1, KMO, KAT, QPRT) and extracellular release of these molecules (e.g., kynurenine, kynurenic acid, NAMPT, quinolinate, NMN, NAM) may be therapeutic targets in CKD (Figs. 3 and 9). These ligands may activate TLR4 (NAMPT, NAPRT) [80], HCAR3 (kynurenic acid) [110], n-methyl-d-aspartate (NMDA: quinolinate receptor) [117], and AHR (kynurenic acid, kynurenine) [20] (Fig. 9). Further exploration of these ligands, their receptors, and downstream responses may provide insight into CKD pathogenesis and therapeutic targets.

Niacin may promote insulin sensitivity and contribute to NAD production (Figs. 3 and 5). Experimentally, NAD metabolites in the salvage pathway (NAM, NMN, NR) antagonize renal tubular necrosis, inflammation, and fibrosis (Figs. 1, 6, 7, 8). Recent study also suggests that administration of NR [130] and NAD [131] may improve anemia, which is a common complication in patients with CKD [18]**.** The mechanisms may be associated with increased activation of sirtuins that antagonize HIF-1α, NF-kB, and AHR activity but promote HIF-2α responses [17, 25, 79] (Figs. 1, 6, 7, 8). NAD metabolite post-translational responses also include increased activation of methyltransferases, reduced activation of PARPs, and changes in phosphorylation and ubiquitination of proteins [25, 88]. Continued research of these NAD-induced post-translational responses in CKD may provide insight into the function of the shared dimer partner, HIF-1β, in the activation of HIF and AHR genes during CKD.

Moreover, post-translational responses may also be initiated through non-coding RNAs (ncRNAs). Cells release ncRNAs in extracellular vesicles, ribonucleoprotein complexes or lipoproteins, particularly HDL [132]. An effect of niacin is to increase HDL and lower LDL levels [33], suggesting that the vitamin may alter ncRNAs. These molecules include microRNAs, long ncRNAs, piwi-interacting RNAs, small nucleolar RNAs, circular RNAs and yRNAs, and are together termed the regulome. They are differentiated by size and function, represent 80% of the 98.8% non-coding genes in humans, and contribute to the pathogenesis of CKD [132].

A long ncRNA, metastasis-associated lung adenocarcinoma transcript 1 (MALAT1), is upregulated in patients with cancer and various lung diseases, including COVID-19 [133]. With respect to kidney disease, circulating levels of MALAT1 are elevated in patients with AKI compared to healthy controls [134]. In mice exposed to 8% O_2_ inspiratory normobaric hypoxia for 24 h, MALAT1 is highly induced in kidney proximal tubule epithelial cells [135]. Hypoxia-exposed (1% O_2_) human-kidney 2 (HK-2) cells promote production of inflammatory cytokines and induce cellular apoptosis, which are both reduced in MALAT1 knockdown experiments [134], suggesting that MALAT1 may have a molecular role in kidney disease. Moreover, in various tumor cell lines, MALAT1 is induced by HIF-1α [136], HIF-2α [135], and AHR [137]. MALAT1 may also inhibit FOXO1-induced SIRT1 transcription [138], reduce insulin sensitivity [139, 140], promote renal fibrosis [141], and induce p53 cell signaling [142], which may indicate that MALAT1 antagonizes the functions of niacin, NAM, NMN, NR, and NAD.

Some therapies that target long ncRNAs include inhibitors of transcription, post-transcription inhibitors, steric hindrance of secondary structure formation, or protein binding interaction inhibitors that are each complicated by the specificity, targeted delivery, and tolerability of the molecules [143]. Inhibition or overexpression of ncRNAs, like MALAT1, may also trigger the production or inhibition of additional ncRNAs in the regulome [134, 144], increasing the need to understand their functions. In a clinical trial, 22 obese men received extended-release niacin or placebo over 8 weeks, revealing an increase in 6 microRNAs out of the 758 tested in human subcutaneous adipose tissue biopsies [145]. In rats fed niacin over 28 days, plasma levels of NAM were elevated and 42 out of 259 miRNAs were differentially expressed compared to controls [146]. Niacin therefore can modulate the regulome. Understanding ncRNA networks associated with niacin, NAM, NMN, NR, and NAD may lead to better therapies that target MALAT1 or additional cell signals altered in CKD.

The use of nanoparticles in the delivery of ncRNA inhibitors may increase specificity and tolerance [147]. Nanoparticles may be a mechanism in targeted delivery of niacin, NAM, NMN, NR, NAD, or siRNA to pathogenic sites. Nanoparticles are nanometer-sized partiles that traverse biologial systems, which are protected by nanometer-sized barriers. Depending on the particle size, nanoparticles can penetrate almost all tissues. As a result, nanoparticles can function as disease biomarkers and drug delivery carriers [148, 149]. In clinical studies of CKD, nanoparticles are increasingly explored to diagnostically determine urinary microalbuminuria, kidney inflammation and fibrosis. Chitosan, polyaniline, superparamagnetic iron oxide, gold, and magnetic are some of the nanoparticles used in drug delivery to treat CKD [149]. Non-toxic magnetic nanoparticles (MNPs) such as magnetite (Fe_3_O_4_) and maghemite (*γ*-Fe_2_O_3_) catalyzed organic processes consume a minimum of energy and minimize waste (a form of green chemistry) and may therefore pose minimum risks and adverse events to human health [150]. MNPs have a relatively large surface that enables binding to and carrying of other compounds to targeted locations in the body using an external magnetic field [148]. Tagging niacin to MNPs has been explored in vitro [150]. Targeted delivery of siRNA through connexin 43 exosome-mimicking chitosan nanoparticles is also being explored [151], which may offer insight into targeting CKD tissue with over-expressed connexin 43 [4] and/or regulating connexin 43 function in cellular NAD retention [6]. Thus, nanoparticle tagged NAD metabolites, ncRNAs, or siRNA targeted to kidney tissue may offer novel approaches slow or halt CKD progression.

## Conclusion

HIFs and AHR contribute to the regulation of metabolism, lipolysis, inflammation, endothelial dysfunction, thrombosis, and tissue injury that variously manifest in complications of tissue ischemia, insulin resistance, thrombosis, and fibrosis in CKD. These responses are associated with the disruption of NAD metabolism. Niacin, a primary NAD metabolite, may protect against insulin resistance through HDL cholesterol production and HCAR receptor cell signals. In various studies, NAM metabolites ameliorated cell signals associated with CKD progression. The mechanisms of this amelioration are primarily associated with increased NAD production that selectively increases SIRT1 activity but inhibits PARP activity. The post-translational modifications associated with these molecules alter NF-κB, HIF-1α, HIF-2α, and AHR cell signals. The complexity of these responses is highlighted by the fact that HIF-1α and AHR share a dimer partner, HIF-1β, that also complexes with HIF-2α. This transcription factor is stabilized by NAD-induced SIRT2 activity and is pivotal in maintaining the production of erythropoietin and NAMPT, which are both dysregulated in CKD. Thus, levels of NAD regulate the activity of these transcription factors and tissue homeostasis.

Cross talk among these networks may include ncRNAs. Further exploration of cell signals (FOXO1, PGC-1α, SIRT1, p53, SMADs, MALAT1) altered in CKD and modulated by ncRNAs and NAD metabolites may offer insight into novel therapeutic approaches. The delivery of ncRNA, siRNA, NAD metabolites or additional molecules through nanoparticles may provide more targeted therapeutic responses. Current therapies involve hypotensive drugs, anti-fibrotic drugs and for particular diseases, immunosuppressive agents. Renin–angiotensin–aldosterone system (RAAS) pathway inhibitors have important anti-fibrotic effects, in addition to their hypotensive effects. These approaches all have value and may be combined. Nonetheless, too many patients with CKD progress to end-stage kidney disease, with attendant morbidity and premature mortality. New biomarkers and new therapeutic approaches, some of which are described here, may help to develop novel agents to slow or even halt progressive loss of kidney function.

## Data Availability

Not applicable.

## Abbreviations

1-MT: 1-Methyl-d-tryptophan
3-HAA: 3-Hydroxy-anthranilic acid
3HK: 3-Hydroxy-l-kynurenine
l AHR: Aryl hydrocarbon receptor
AKI: Acute kidney injury
ALDOA: Aldolase
AOX: Aldehyde oxidase
ARNT: AHR nuclear translocator
cADPR: Cyclic ADP ribose
CKD: Chronic kidney disease
CLP: Cecal ligation and puncture
CPT1A: Palmitoyltransferase 1A
CREB: CAMP-response element binding protein
Dnmt1: DNA methyltransferase-1
ENOL: Enolase
eNAMPT: Extracellular NAMPT
eNAPRT: Extracellular NAPRT
ENT1: Equilibrative nucleoside transporter 1
eGFR: Estimated GFR
FKBP: FK-506 binding protein
FSGS: Focal segmental glomerulosclerosis
GFR: Glomerular filtration rate
GLUT1: Glucose transporter
GSK-3β: Glycogen synthase kinase-3
HCAR: Hydroxy-carboxylic acid receptors
HDL: High-density lipoprotein
HIF: Hypoxia-inducible transcription factor
HK2: Hexokinase 2
IDO1: Indoleamine 2, 3-dioxygenase 1
I/R: Ischemia and reperfusion
KAT: Kynurenine aminotransferase
KMO: Kynurenine 3-monooxygenase
Kyn: Kynurenine
LAT1: l-type amino acid transporter
LDHA: Lactate dehydrogenase A
LDL: Low-density lipoprotein
MAVS: Mitochondrial antiviral-signaling protein
MCT1: Monocarboxylate transporter 1
MNA: N1-methylnicotinamide
MNP: Magnetic nanoparticles
NAD: Nicotinamide adenine dinucleotide
NADSYN1: NAD synthetase 1
NAM: Nicotinamide
NAMN: Nicotinic acid mononucleotide
NAMPT: Nicotinamide phosphoribosyltransferase
NAPRT: Nicotinic acid phosphoribosyltransferase
ncRNA: Non-coding RNA
N-Me-2PY: n-methyl-2-pyridone-5-carboxamide
N-Me-4PY: n-methyl-4-pyridone-3-carboxamide
NMN: Nicotinamide mononucleotide
NMNAT: Nicotinamide mononucleotide adenylyltransferases
NNMT: Nicotinamide n-methyltransferase
NR: Nicotinamide riboside
PARP: Poly-ADP ribose polymerase
PDE: Phosphodiesterase
PDK1: Pyruvate dehydrogenase 1
PGC-1α: Peroxisome proliferator-activated receptor-gamma coactivator
PGK1: Phosphoglycerate kinase-1
PHD: Prolyl hydroxylase domain
PPARA: Peroxisome proliferator activated receptor alpha
QPRT: Quinolinate phosphoribosyl transferase
RIG-I: Receptor retinoic acid-inducible gene I
SAH: S-adenosylhomocysteine
SAM: S-adenosylmethionine
SIRT: Sirtuin
SMCT: Sodium-coupled monocarboxylate transporter
TDO: Tryptophan dioxygenase
TLR4: Toll-like receptor 4
UUO: Unilateral ureteral obstruction
VHL: Von Hippel-Lindau
